# The Role of Transaxemic Acid in Plastic Surgery: An Expert Video Roundtable Discussion

**DOI:** 10.1093/asjof/ojaf091

**Published:** 2025-07-09

**Authors:** Jeffrey Kenkel, James Zins, James Fernau, Gerald O’Daniel

The authors are pleased to present an expert roundtable discussion on transaxemic acid (TXA) in plastic surgery (Video). The discussants include a panel of leading experts in aesthetic surgery, and the video roundtable was recorded live at The Aesthetic MEET 2025 in Austin, TX. The panelists began by discussing the clinical uses of TXA, including its anti-inflammatory effect, prevention of platelet aggregation, and its ability to prevent drainage during procedures, which saves surgeons time in the operating room. They mentioned the benefits of TXA and its low complication rate, and its great potential in surgery compared with epinephrine, which some patients may be sensitive to. The panelists discussed their uses of TXA in their own practices and how they have adjusted dose and application based on their experiences in the operating room. They concluded by agreeing that more studies with larger numbers of patients are needed to confirm some of the anecdotal reports of TXA's uses.

